# Refractory Status Epilepticus Associated With a Pathogenic Variant in TNFRSF13B

**DOI:** 10.7759/cureus.48222

**Published:** 2023-11-03

**Authors:** Jennifer H Yang, Nicholas Scanlon, Wonhee Woo, Jamie N LaBuzetta, Cynthia Gonzalez, Lori Broderick, Taylor Doherty, Marc Riedl, Anastasie Dunn-Pirio

**Affiliations:** 1 Neurosciences, University of California San Diego, San Diego, USA; 2 Pediatric Neurology, Rady Children's Hospital San Diego, San Diego, USA; 3 Allergy and Immunology, University of California San Diego, San Diego, USA; 4 Allergy and Immunology, Kaiser Permanente San Jose Medical Center, San Jose, USA

**Keywords:** primary immunodeficiency disease, autoimmune epilepsy, febrile status epilepticus, refractory status epilepticus, common variable immunodeficiency deficiency

## Abstract

Febrile infection-related epilepsy syndrome (FIRES) is a rare epileptic syndrome characterized by new-onset refractory status epilepticus preceded by a febrile illness. Limited literature exists regarding the relationship between primary immunodeficiencies and immune-mediated epilepsy, and the relationship between new-onset refractory status epilepticus and common variable immunodeficiency (CVID) is not well-understood. We present a case of a 21-year-old female with a history of recurrent sinus infections, asthma, thrombocytopenia, atrioventricular nodal reentrant tachycardia, and neonatal seizures who presented with fever and new-onset status epilepticus. She was ultimately diagnosed with a heterozygous variant in *TNFRSF13B* c.311G>A (p.Cys104Tyr), which encodes for a tumor necrosis factor receptor implicated in CVID.

## Introduction

Febrile infection-related epilepsy syndrome (FIRES) is a rare epileptic syndrome characterized by new-onset refractory status epilepticus (NORSE) preceded by a febrile illness [1]. Studies have shown that elevated levels of pro-inflammatory cytokines, including IL-6, may be involved [1], but limited literature exists regarding the relationship between primary immunodeficiencies and immune-mediated epilepsy. Neurological complications of common variable immunodeficiency (CVID) are rare. The relationship between seizures and CVID is not well understood, particularly with the phenotypic heterogeneity in CVID-associated genes [2]. To our knowledge, none have been reported in association with FIRES/NORSE [3].

This article was previously presented as a meeting abstract at the 2022 AAN Annual Meeting on April 2, 2022.

## Case presentation

**Figure 1:**
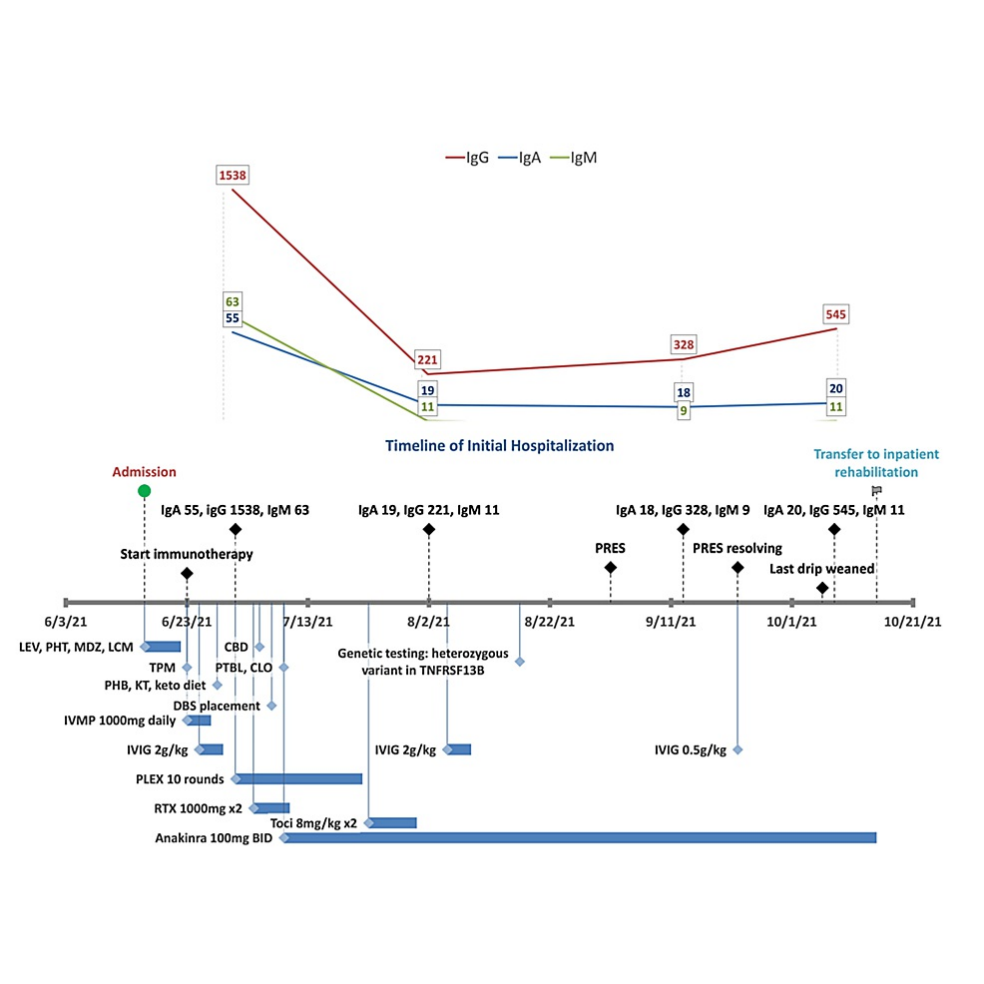
Timeline of initial hospitalization and timing of immunoglobulin levels with medication administration and duration Abbreviations: LEV = levetiracetam, PHT = phenytoin, MDZ = midazolam, LCM = lacosamide, TPM = topiramate, PHB = phenobarbital, KT = ketamine, keto = ketogenic, PTBL = pentobarbital, CLO = clobazam, CBD = cannabidiol, DBS = deep brain stimulation, IVMP = intravenous methylprednisolone, IVIG = intravenous immunoglobulin, PLEX = plasma exchange, RTX = rituximab; Toci = tocilizumab; immunoglobulins in mg/dL

**Figure 2:**
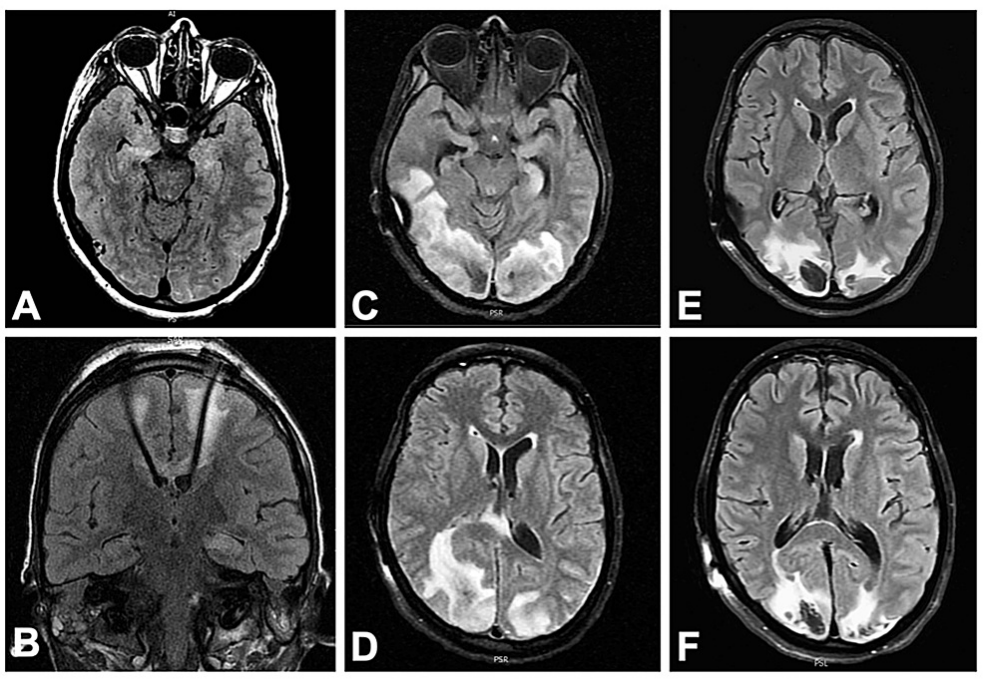
Evolution of neuroimaging A: T2 hyperintensity of bilateral mesial temporal lobes on admission; B: Placement of deep brain stimulator with electrode tips terminating in anterior thalami nuclei; C,D: Confluent posterior predominant, asymmetric subcortical and deep white matter hyperintensity and edema consistent with PRES; E,F: Repeat imaging two weeks later showing resolving prior T2 lesions and development of intraparenchymal hematomas

A 21-year-old female presented to the neurocritical care unit with three days of headache, fever, and lethargy followed by new onset status epilepticus consistent with FIRES/NORSE. She had a history of recurrent childhood sinus infections, asthma, thrombocytopenia of unclear etiology, atrioventricular nodal reentry tachycardia, neonatal seizures, and recurrent aseptic meningitis in adolescence. Her initial cerebrospinal fluid (CSF) studies were unremarkable, but they later demonstrated a lymphocytic pleocytosis of 37 cells/uL, an elevated total protein of 124 mg/dL, elevated IL-6 (13.5 pg/ml; normal ≤7.5), and elevated IL-2R (soluble: 64.3 pg/ml; normal ≤26.8). A subsequent CSF analysis demonstrated further IL-6 elevation of 48.8 pg/ml. A brain magnetic resonance imaging (MRI) showed T2 hyperintensities in the mesial temporal lobes concerning limbic encephalitis. However, a comprehensive evaluation for known antibody-mediated autoimmune and paraneoplastic causes of encephalitis was negative except for a low serum glutamic acid decarboxylase (GAD65) antibody titer of 0.25. Diagnostic workup for other inflammatory white matter disorders, rheumatological disease, and infectious causes was negative. During her hospital course, she received multiple antiseizure medications, treatment with the ketogenic diet, bilateral deep brain stimulator (DBS) placement in the anterior thalami, and multiple immunotherapies including intravenous methylprednisolone, intravenous immunoglobulin (IVIG), plasmapheresis, rituximab, tocilizumab, and anakinra (Figure 1). She had a prolonged hospitalization complicated by various nosocomial infections and posterior reversible encephalopathy syndrome (PRES) that improved over the course of several weeks (Figure 2).

During her diagnostic workup, she was noted to have a low IgA level of 55 mg/dL (normal 70-400 mg/dL) with a normal IgG level of 1538 mg/dL and IgM of 63 mg/dL, although these levels were obtained one week after she received IVIG 2 g/kg (Figure 1). Unfortunately, a baseline IgG level was not obtained prior to IVIG administration. After receiving two doses of IV rituximab 1000 mg for presumed autoimmune encephalitis, she developed pan-hypogammaglobulinemia with an IgA level of 19 mg/dL, IgG of 221 mg/dL, and IgM of 11 mg/dL with normal IgG subclasses, raising the suspicion for an underlying primary immunodeficiency. Genetic testing revealed a pathogenic heterozygous variant in *TNFRSF13B* c.311G>A (p.Cys104Tyr). She was started on monthly 0.5 g/kg IVIG, which was held for one month after she developed PRES. She was eventually discharged to an inpatient rehabilitation facility and gradually improved in her motor, fine motor, and speech functions.

Currently, she is 18 months post-discharge, and her seizures remain refractory but improved on several antiseizure medications, DBS use, and anakinra 100 mg subcutaneously three times a day. She has daily short focal onset seizures consisting of gaze deviation and altered awareness. However, her seizures worsened when anakinra was reduced to twice-a-day dosing with the same antiseizure medication dosing and DBS settings. Seizures were reduced again when anakinra was increased back to three times a day. She continued to have undetectable IgM levels <5, low IgA 19, and IgG 474 mg/dL, resulting in frequent infections. Therefore, she is currently on a regimen of 0.5 g/kg IVIG every three weeks. B cell subsets demonstrate continued B cell suppression 15 months after rituximab with 0.4% CD19+ B cells. Thus, further analysis of B subsets was unattainable.

## Discussion

The *TNFRSF13B* gene encodes for transmembrane activator and calcium modulator and cyclophilin ligand interactor (TACI), which is a tumor necrosis factor receptor expressed on marginal zone B cells, plasma cells, and CD27+ memory B cells. TACI promotes T cell-independent antibody responses and plasma cell differentiation and counteracts BAFF-driven B cell activation, affecting isotype switching and immunoglobulin production [4-6]. Studies of TACI deficiency in humans and mouse models demonstrate its important role in toll-like receptor (TLR) pathways [7-10] and the generation of autoreactive B cells [11]. The Cys104Tyr (C104Y) variant found in our patient is a missense variant reported among a cohort of patients with *TNFRSF13B* variants [12]. The cysteine residue at this amino acid position is highly conserved, with an allele frequency of 0.00016, and no homozygotes, on gnomAD. Functional algorithms with advanced modeling in protein sequence and biophysical properties (SIFT, poly-phen2, Align-GVGD) predict this variant to be probably damaging and deleterious to protein function. This variant is reported in individuals with CVID and IgA deficiency in eight different publications [12-19]. Notably, the Cys104Arg (C104R) variant at the same amino acid position, along with A181E, is one of the most common variants associated with CVID [12,15].

Biallelic/homozygous *TNFRSF13B* mutations are reported in hypogammaglobulinemia in children, and monoallelic/heterozygous variants are implicated in autoimmune disease in adulthood, although clinical heterogeneity exists even within the same family [11,13]. In a cohort study of 50 patients with *TNFRSF13B*-associated CVID, patients with heterozygous variants (the most common being C104R) had a higher percentage of autoimmunity compared to control participants without TACI mutations [12]. One study of B cell function from patient-derived biallelic and monoallelic variants in TACI compared to healthy controls demonstrated that TACI plays an important role in B cell tolerance [11]. Only participants with a heterozygous variant in TACI demonstrated impaired immune tolerance and secreted high levels of antinuclear antibodies along with increased circulating B cell lymphoma 6-expressing T follicular helper cells [11]. This suggests that partial TACI protein function in patients with one mutation results in autoimmunity because of impaired removal of autoreactive B cells at a central B cell checkpoint. The most common autoimmune disease in TACI-heterozygous patients was autoimmune thrombocytopenia, a condition that our patient may have developed in childhood. Of the 45 patients with B cell subsets, CD19+ B cell counts were normal in approximately half the patients, but IgD−IgM−CD27+ switched memory B cells were low in 76% of the patients. This decrease in class-switched memory B cells was not reported in patients with biallelic or compound heterozygous mutations. Interestingly, patients with biallelic TACI mutations did not demonstrate autoimmunity, a finding that is consistent with an Italian cohort of 189 CVID patients [12,14]. The Italian study also reported a higher frequency of switched memory B cells compared to heterozygous patients [14].

Clinically, CVID exhibits phenotypic and genetic heterogeneity, including variable severity in the degree of immunodeficiency as well as immune dysregulation and autoimmunity. While many other primary immunodeficiencies exhibit higher rates of monogenic forms, only 2-10% of CVID are monogenic, and a larger subset of CVID likely has complex, rather than monogenic, inheritance [2]. Genes implicated in monogenic CVID include *ICOS*, *TNFRSF13B* (TACI), *TNFRSF13C* (BAFF-R), *TNFSF12* (TWEAK), *CD19*, *CD81*, *CR2* (CD21), *MS4A1* (CD20), *TNFRSF7* (CD27), *IL21*, *IL21R*, *LRBA*, *CTLA4*, *PRKCD*, *PLCG2*, *NFKB1*, *NFKB2*, *PIK3CD*, *PIK3R1, VAV1, RAC2, BLK, IKZF1* (IKAROS), and *IRF2BP2* [2].

Neurological complications related to *TNFRSF13B* mutations are not well understood. However, neurological involvement in CVID includes CNS infections, polyneuropathy, vasculitis, and transverse myelitis [20]. Limited literature exists regarding the relationship between CVID and autoimmune epilepsy. The presence of GAD65 antibodies in our patient’s serum is likely nonspecific as neurological complications from GAD65 antibodies occur at high titers with antibody positivity in the CSF as well as serum [21]. Interestingly, our patient’s seizure control improved after the addition of anakinra, which may be unrelated to the TACI mutation. However, the TACI heterozygous variant may have predisposed her to a cascade of immune dysregulation following a febrile illness.

Given the complex nature of CVID and epilepsy genetics, the *TNFRSF13B* variant was likely a susceptibility factor for CVID. There were many confounding variables in this case related to treatment including plasma exchange, rituximab, and antiseizure medications. Other factors including gene-gene and gene-environmental interactions likely contributed to the clinical severity of this case. Larger patient cohort studies and functional modeling for this gene are needed to evaluate the potential causal relationship between TACI gene mutations and seizure risks. While there is a broad spectrum in the clinical presentation of *TNFRSF13B*-associated disease, this diagnosis should still be considered in cases of new-onset refractory status epilepticus with otherwise negative workup for other differential diagnoses. Careful screening prior to initiating immunotherapies such as IVIG and B cell depleting therapy should be performed including quantitative immunoglobulins and lymphocyte subsets.

## Conclusions

Mutations in *TNFRSF13B* are associated with CVID. Biallelic variants in TACI are associated with hypogammaglobulinemia, and heterozygous variants are associated with autoimmune disease. Neurological complications of CVID are rare. Further studies are needed to investigate the causal relationship between CVID and epilepsy given the many potential confounding variables. However, primary immunodeficiencies should be considered in FIRES/NORSE cases, as immunosuppressive medications such as rituximab can unmask an underlying immunodeficiency. Care should be taken in these cases to select the appropriate immunomodulatory therapies.
